# Distribution and asymptotic behavior of the phylogenetic transfer distance

**DOI:** 10.1007/s00285-019-01365-0

**Published:** 2019-04-29

**Authors:** Miraine Dávila Felipe, Jean-Baka Domelevo Entfellner, Frédéric Lemoine, Jakub Truszkowski, Olivier Gascuel

**Affiliations:** 1 Unité Bioinformatique Evolutive, C3BI, USR 3756, Institut Pasteur & CNRS, Paris, France; 2 Biosciences eastern and central Africa (BecA-ILRI Hub), International Livestock Research Institute, PO Box 30709, Nairobi, 00100 Kenya; 3 Hub Bioinformatique et Biostatistique, C3BI, USR 3756, Institut Pasteur & CNRS, Paris, France; 4 Méthodes et Algorithmes pour la Bioinformatique, IBC - LIRMM, UMR 5506, Université de Montpellier & CNRS, Montpellier, France

**Keywords:** Phylogenetic trees, Distances between bipartitions and phylogenies, R-distance, Random phylogenies, Concentration inequalities, Lattice paths, Primary 92-08, 62P10, 62F40, 62F05, 62F35, Secondary 05A05, 05C05, 60E15

## Abstract

The *transfer distance* (TD) was introduced in the classification framework and studied in the context of phylogenetic tree matching. Recently, Lemoine et al. (Nature 556(7702):452–456, 2018. 10.1038/s41586-018-0043-0) showed that TD can be a powerful tool to assess the branch support on large phylogenies, thus providing a relevant alternative to Felsenstein’s bootstrap. This distance allows a *reference branch*$\beta$ in a reference tree ${\mathcal {T}}$ to be compared to a branch *b* from another tree *T* (typically a bootstrap tree), both on the same set of *n* taxa. The TD between these branches is the number of taxa that must be transferred from one side of *b* to the other in order to obtain $\beta$. By taking the minimum TD from $\beta$ to all branches in *T* we define the *transfer index*, denoted by $\phi (\beta ,T)$, measuring the degree of agreement of *T* with $\beta$. Let us consider a reference branch $\beta$ having *p* tips on its light side and define the *transfer support* (TS) as $1 - \phi (\beta ,T)/(p-1)$. Lemoine et al. (2018) used computer simulations to show that the TS defined in this manner is close to 0 for random “bootstrap” trees. In this paper, we demonstrate that result mathematically: when *T* is randomly drawn, TS converges in probability to 0 when *n* tends to $\infty$. Moreover, we fully characterize the distribution of $\phi (\beta ,T)$ on caterpillar trees, indicating that the convergence is fast, and that even when *n* is small, moderate levels of branch support cannot appear by chance.

## Introduction

The *transfer distance* or *R-distance* was introduced in the classification framework by Day (1981) and Régnier (1965), as a measure of (dis)similarity between partitions of a set. It is defined as the minimum number of elements that need to be transferred from one class to another (or removed), in order to transform one partition into the other. This distance possesses some desirable properties, for example its low computational cost in comparison with other metrics, as established by Day (1981). Charon et al. (2006) studied other characteristics of this distance such as the maximum transfer distance that can be obtained when comparing two partitions with a fixed, but possibly different, number of classes. As highlighted by Denœud (2008), it proves challenging to study the theoretical properties of the transfer distance, so the author used simulations to approximate its distribution and mean on random partitions.

The interest in using the transfer distance to compare phylogenetic trees started with a seminal paper by Day (1985). In the field of computational biology, problems involving tree comparison have remained a major challenge for many years. A common concern is to define biologically meaningful metrics on trees. The transfer distance is a measure to compare bipartitions, and a phylogenetic tree is unambiguously defined by the set of bipartitions induced by its branches. Then, a logical question to ask is whether we can define a metric on trees based on this transfer distance on bipartitions. However, there are multiples ways to define such a metric. Day (1985) proposes several algorithms and methods to solve related tree problems, in particular the construction of tree consensus. As discussed by Day (1985), this task requires the optimization of a consensus index, which can be defined using the transfer distance or other metrics, such as the well-known Robinson–Foulds (RF) metric (Robinson and Foulds 1981). The latter is probably the most widely used distance between trees and is defined as the number of bipartitions belonging to one tree but not to the other. However, the RF metric is known to have several drawbacks, including its lack of robustness, since it is highly sensitive to small tree changes, as pointed out by Lin et al. (2012) and Bogdanowicz and Giaro (2012).

Boc et al. (2010) used RF and a transfer-based dissimilarity to compare gene trees to species trees and detect horizontal gene transfers. They showed that the transfer approach provides better results than RF. However, their transfer-based dissimilarity is not a metric, since it violates the triangle inequality in some cases (Boc et al. 2010, p. 197, Proposition 1). Lin et al. (2012) addressed this problem using the minimum-cost matching between the two sets of bipartitions induced by both trees. For that metric, also relying on the transfer distance, the triangle inequality holds. Moreover, Lin et al. (2012) proposed a low-polynomial time algorithm to compute this new tree metric and demonstrated its robustness compared to RF.

Recently, we proposed a new bootstrap method for large phylogenetic trees that relies on branch comparisons based on the transfer distance (Lemoine et al. 2018). The aim was to use a more fine-grained measure for the presence of a branch in a tree, rather than the binary values used in Felsenstein’s classical phylogenetic bootstrap technique (Felsenstein 1985). In this approach, we compare a *reference branch*$\beta$ in a *reference tree*${\mathcal {T}}$ to another tree *T*, typically a bootstrap tree, by taking the minimum of the transfer distance from $\beta$ to any branch *b* in *T*, which is called the *transfer index* and denoted by $\phi (\beta ,T)$. Next, the average of $\phi$ over a set of bootstrap trees is used to define, after appropriate normalization, the so-called *transfer bootstrap expectation* (TBE). We explored the behavior of TBE as a measure of support for the branches of a phylogenetic tree, compared to that of Felsenstein’s support (FS). In a number of experiments using both real and simulated data, we found that TBE outperformed FS. This was particularly noticeable for deep branches and large values of *n*, where FS often failed to detect the phylogenetic signal in the trees. Unstable taxa are inevitable (sequence and reconstruction errors, recombination, etc.) under these conditions, and nearly correct branches are seen as absent by the standard bootstrap, thus yielding low support values, while TBE is able to detect the few misplaced taxa and provides high support values for those branches. In view of those results, TBE shows promise as a useful tool in phylogenetic analysis. Lemoine et al. (2018) studied and discussed several of its properties, but there was still a need for further mathematical work to fully understand the transfer index and TBE. The main motivation for the present work is therefore to study the properties of the transfer index and support, and characterize their asymptotic behavior when the reference branch is compared to a tree *T* drawn randomly according to some null model, reflecting in a bootstrap context the absence of phylogenetic signal in the analyzed data set.

To be more specific about the results obtained here, let us fix $n\ge 4$ and consider phylogenetic trees on a set *X* of *n* taxa. To distinguish the two sides of the reference bipartition $\beta$, we say that its *light side* contains $p \ge 2$ taxa while its *heavy side* has $n-p \ge p$ taxa. The TBE proposed by Lemoine et al. (2018) is the average, over all the bootstrap trees, of the *transfer support* function (TS), which is defined as $1 - \phi (\beta ,T)/(p-1)$. Equivalently, we can compute the average of $\phi (\beta , T)$ over all boostrap trees and apply the same linear normalization to obtain TBE. It is not hard to see that $0\le \phi (\beta ,T)\le p-1$ and thus $\text {TS}\in [0,1]$.

**Fig. 1:**
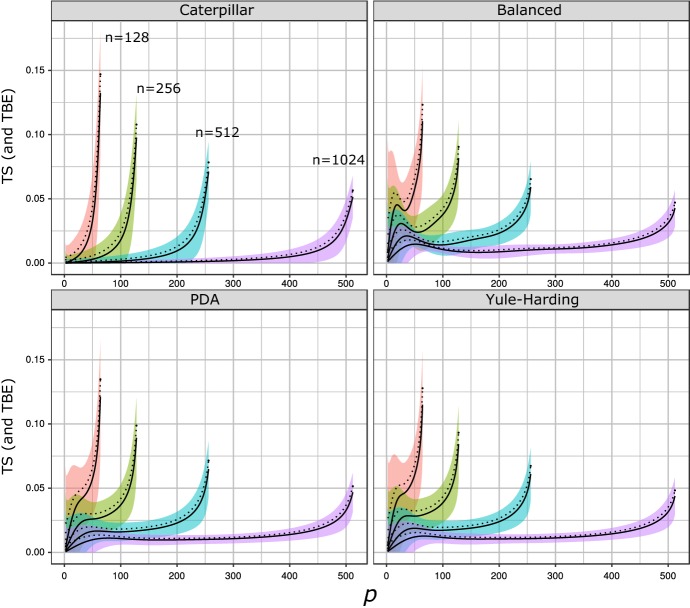
Simulations of TS and TBE for random trees. The four panels correspond to the four tree models: caterpillar, fully balanced, PDA, and Yule-Harding. For each of these models, we considered 4 different values of *n* and used the following color codes to distinguish them: 128 (red), 256 (green), 512 (blue), and 1024 (purple). For each model and for each value of *n* and *p* (from 2 to ${\lfloor }{n/2}{\rfloor }$), we randomly drew 100 reference bipartitions and 1000 trees to be compared to the reference bipartition. We plotted against *p* the mean of the $100\times 1000$ TS values obtained for each *n* and for each model (bold lines), and the standard deviation $\sigma$ observed among these 100,000 values (shaded areas). The mean value for TS also corresponds to the mean value of TBE, assuming the corresponding null model (TBE is obtained by averaging TS among the “bootstrap” trees). We also plot (dashed line) that mean value plus $3.09 \times \sigma /10$, corresponding to a one sided p-value of $10^{-3}$ to reject the null model with 100 bootstrap replicates (see Sect. 6). We see from these results that not only is TS close to 0 (e.g. $\sim 0.05$ or less when $n = 1024$), but also that the standard deviation is small, especially with large *n*. Moreover, even with moderate $n = 128$, TS remains relatively close to 0 from a branch support perspective, and TBE $= 0.15$ is sufficient to reject the null model with 100 replicates (color figure online)

Importantly, computer simulations showed that $\text {TS}(\beta ,T)$ converges towards zero when *T* is random. In other words, the branch support is null in the absence of phylogenetic signal, while it is equal to 1 when all bootstrap trees contain the reference branch. These two properties are highly desirable for defining a meaningful branch support. However, the convergence to zero with random“bootstrap” trees is somewhat surprising, as it means that the expected value of $\phi (\beta ,T)$ converges to its upper bound, $p-1$, for large random trees. To illustrate this finding, we reproduce in Fig. 1 the results obtained from computer simulations by Lemoine et al. (2018), where we looked at four random tree models for *T*. We considered (1) two simple, extreme cases for the topology of *T*: caterpillar trees and perfectly balanced binary trees; and (2) two classic random tree models: the Proportional to Distinguishable Arrangements (PDA) and Yule-Harding models (see Sect. 2 for further details). For each of these models, we performed simulations for different values of *n* (128, 256, 512, and 1024) and all the possible values of *p* for each *n* (i.e. $2\le p\le {\lfloor }{n/2}{\rfloor }$). Then, for each value of (*n*, *p*) and for each model, we randomly generated a set of reference bipartitions and a set of random trees to be compared to the reference bipartitions. Results are shown in Fig. 1, where we plotted the TS values thus obtained against the different values of *p*. We see striking evidence that, on average, TS stays close to 0 with random trees, so the transfer index stays close to its upper bound $p-1$ for all *p*. Additionally, we observe that the maximum value of the TS attained over all possible values of *p* seems to be obtained at $p={\lfloor }{n/2}{\rfloor }$ and to decrease when *n* increases. Moreover, this asymptotic behavior seems to be independent of the topology/model considered for the tree *T*. These initial observations justify the simple normalization by $p-1$ proposed by Lemoine et al. (2018) and motivate the questions we address here.

Our first result consists in the characterization of the asymptotic behavior of the transfer index $\phi (\beta ,T)$ for a random bipartition $\beta$ of the set *X* and any given tree *T*. We prove that the transfer index converges in probability to $p-1$ when *p* is fixed (or grows slower than $\sqrt{n}$) and *n* tends to $\infty$. The proof relies on the comparison of the transfer index with the parsimony score of a binary character, and a result from (Steel and Penny 2005). We then use concentration inequalities to characterize the asymptotic behavior of the transfer index when *p* grows faster, depending on *n* (e.g. when $p = {\lfloor }{n/2}{\rfloor }$). Lastly, when *T* is a caterpillar tree, we fully characterize the probability distribution of the transfer index based on a one-to-one correspondence between these trees and North-East (NE) lattice paths, a common technique for counting combinatorial objects (Mohanty 1979). All of these results show that $p-1$ is the appropriate normalization constant for the TS and TBE, as proposed by Lemoine et al. (2018).

The paper is organized as follows. In Sect. 2, we give the main definitions and properties of the concepts described earlier. Section 3 is devoted to the results concerning the parsimony score, and Sect. 4 presents the asymptotic results using concentration inequalities. Details on the specific case of the caterpillar tree are given in Sect. 5. In Sect. 6, we discuss the impact of our findings on the phylogenetic bootstrap, and propose several conjectures and directions for further work.

## Preliminaries

In this section, we give the main definitions and general properties on phylogenetic trees that are needed for the rest of the paper. We refer to Semple and Steel (2003) for an extensive mathematical treatment of this subject.

Let us fix $n\ge 4$ and *X*, a set of *n* taxa. We consider phylogenetic trees on *X*, that is, trees whose leaves are mapped one-to-one to *X*. These trees are called *phylogeneticX-trees* or simply phylogenies. For simplicity of notation, we shall always take $X = \{1,2,\ldots ,n\}$. Denote by $\text {UB}(n)$ the set of all unrooted *binary* phylogenetic trees (every interior vertex has degree 3) on *n* leaves. For a phylogenetic tree *T*, we use ${\mathcal {E}}(T)$, ${\mathcal {V}}(T)$ to denote respectively the set of edges (or branches) and the set of vertices (or nodes) of the tree.

For any *X*-tree *T*, a branch $b\in {\mathcal {E}}(T)$ can be encoded in several equivalent ways, that we will use indistinctly depending on the context. First, any branch *b* defines a bipartition (or split), and we can associate *b* to a vector *v*(*b*) in $\{0,1\}^n$ by assigning the same number (e.g. 0) to all the elements on the same side of the split induced by this branch. Notice that *b* is also encoded by ${{\overline{v}}}(b)$, the negation of *v* (i.e. the 0 values are turned into 1 and vice versa). Likewise, we can identify a bipartition, with a *bicoloration* of the leaves, that is a function that assigns one of two colors (black $={\mathbf{B }}$ or white $={\mathbf{W }}$) to each leaf label. Notice however, that we can consider a bipartition or a bicoloration on a tree that does not correspond to any branch in this tree. To make the distinction, we say $b\in {\mathcal {E}}(T)$ for the bipartitions induced by branches on the tree *T*, and we use ${\mathcal {X}}:=\{\chi :X\rightarrow \{{\mathbf{B }},{\mathbf{W }}\} \}$ to denote the set of all possible bicolorations of the tips of *T*. Then, an element of ${\mathcal {X}}$ does not necessarily correspond to a branch in *T*, but to a bicoloration of its tips.

**Fig. 2:**
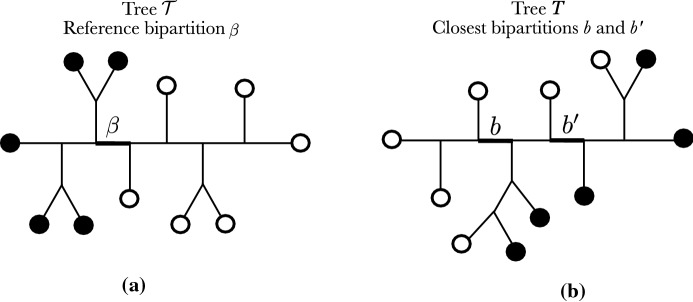
**a** An example of a reference branch $\beta$ dividing *X* in $p=5$ black tips, and $n-p=6$ white tips. **b** Another *X*-tree *T* in which the closest bipartitions to $\beta$ are *b* and $b'$, both giving a transfer distance $\delta (\beta ,b)=\delta (\beta ,b')=3$, and thus $\phi (\beta ,T) = 3$

We will leverage the visual aspect of the two-color representation and, throughout the rest of the article, we associate the *p* taxa of the light side in the reference bipartition with the black color ${\mathbf{B }}$ and the $n-p$ ($\ge p$) taxa of the heavy side with the white color ${\mathbf{W }}$ (Fig. 2, left). The set of bicolorations satisfying that $|\{i\in X:f(i)={\mathbf{B }}\}|=p$ will be denoted by ${\mathcal {X}}_p$. A tree *T* endowed with a bicoloration is called a bicolored tree.

As described in the Introduction, the transfer distance is used to compare a branch $\beta$ in the reference phylogeny ${\mathcal {T}}$, to a second branch *b* in another phylogeny *T*, both on the same taxa set *X*. This distance can easily be defined using the Hamming distance *H* between two vectors of equal size.

### Definition 1

*(Transfer distance)*

$$\begin{aligned} \delta (\beta ,b) :=\min \{H\left( v(\beta ),v(b)\right) , H\left( {{\overline{v}}}(\beta ), v(b)\right) \}. \end{aligned}$$

Based on this definition, notice that $\delta (\beta ,b)=0$ if and only if $\beta$ and *b* define the same bipartition. To measure the degree of presence of $\beta$ in *T*, we define the *transfer index*, denoted by $\phi (\beta ,T)$, which is the minimum of the transfer distance over all branches in *T* (Lemoine et al. 2018).

### Definition 2

*(Transfer index)*

$$\begin{aligned} \phi \left( \beta ,T \right) :=\min \limits _{b\in {\mathcal {E}}(T)} \delta (\beta ,b). \end{aligned}$$

As mentioned before, we are interested in the case where the reference tree ${\mathcal {T}}$ and a branch $\beta$ on this tree are fixed. The core idea proposed by Lemoine et al. (2018) is to measure the presence of this reference branch in a set of bootstrap trees by using the following *transfer support* function.

### Definition 3

*(Transfer support)*

$$\begin{aligned} \text {TS}\left( \beta ,T \right) :=1 - \dfrac{\phi (\beta ,T)}{p-1}. \end{aligned}$$

The transfer index and support functions satisfy simple properties that are included here for completeness:

(i) $$\phi \left( \beta ,T \right) = 0 \Longleftrightarrow \beta \in T$$,
(ii) $\phi \left( \beta ,T \right) \in [0,p-1]$ or equivalently $\text {TS}\left( \beta ,T \right) \in [0,1]$.

The first statement can be deduced directly from the definition of $\delta (\beta ,b)$, which is 0 if and only if $v(\beta )=v(b)$ or ${\overline{v}}(\beta ) = v(b)$. Thus, $\phi \left( \beta ,T \right) = 0$ if and only if we can find the bipartition induced by $\beta$ in *T*. Moreover, we say that a bipartition is trivial when it has a single leaf on one side, and the remaining $n-1$ leaves on the other. For a trivial bipartition *b* defined by a taxon that belongs to the light side in $\beta$, we obtain that $\delta (\beta ,b) = p-1$, and since $\phi$ is defined as the minimum taken over all possible branches in *T*, we obtain the statement (ii).

### Null models

The aim of this study is to characterize the distribution and the asymptotic behavior of the transfer index and transfer support when the reference bipartition $\beta$ is compared to a binary phylogenetic *X*-tree *T* that follows a certain null model. We are interested mainly in unrooted trees, but it should be noted that the existence of a root has no influence on transfer distance values: both branches adjacent to the root define the same bipartition.

There are two ways to define the probabilistic models we are considering. First, we can suppose that we have a fixed bicoloration $\chi _p\in {\mathcal {X}}_p$ and that we draw a tree *T* from $\text {UB}(n)$, following some specific probabilistic model. Another way is to consider that the tree is fixed and a bicoloration of its tips is uniformly chosen from ${\mathcal {X}}_p$. In the first case, an interesting question is to consider the probabilistic models that are most commonly used in the field of phylogenetics, such as the Yule-Harding or PDA models. For a fixed tree, a natural question is to look at the two extreme cases for the topology regarding balance. The most imbalanced tree is called the caterpillar tree, which is defined as a binary phylogenetic tree for which the induced subtree on the interior vertices forms a path graph (if the tree is rooted, then the root is at one end of the path). On the other side, we have perfectly balanced trees, that is rooted binary phylogenetic trees with $n = 2^h$ leaves (for some $h\in {\mathbb {N}}$), each of which is at a distance of exactly *h* edges from the root. We refer to Semple and Steel (2003) and Steel (2016) for further details on these tree models.

As explained in the Introduction, we performed computer simulations for these four models to exhibit their asymptotic properties. In Fig. 1, we observe that the asymptotic behavior of the TS seems to be independent of the model considered. This is explained in the following sections. Then, a full theoretical treatment is carried out for the caterpillar tree topology.

## Comparing the transfer index to the parsimony score

We are now interested in comparing the transfer index to the widely used parsimony score introduced by Farris (1970), Fitch (1971), and Hartigan (1973). We show that the transfer index is lower-bounded by the parsimony score minus one, and use this result to obtain our first characterization of the asymptotic behavior of the transfer index.

### Definition 4

*(Parsimony score)* Consider a phylogenetic tree *T* and a bicoloration of its tips $\chi \in {\mathcal {X}}$. Consider an extension of this bicoloration to all the nodes in *T* and denote it by ${{\overline{\chi }}}$ (each internal node is also assigned one of the two colors). Define

$$\begin{aligned} \text {ps}\left( \chi ,T\right) :=\min \limits _{{{\overline{\chi }}}} \sum \limits _{b\in {\mathcal {E}}(T)} \text {diff}(b,{{\overline{\chi }}}), \end{aligned}$$

where $\text {diff}(b,{{\overline{\chi }}})$ is 0 if both nodes connected by *b* have the same color in the extension ${{\overline{\chi }}}$, and 1 otherwise. The extensions reaching the minimum in the above equation correspond precisely to the ancestral reconstruction of internal node coloration, as obtained using Fitch-Hartigan and related algorithms.

By using a simple argument, one can prove the following result from Lemoine et al. (2018), given here for the sake of completeness.

### Lemma 1

For any given *X*-tree *T* and any bicoloration $\chi \in {\mathcal {X}}$, we have that

1 $$\begin{aligned} \text {ps}\left( \chi ,T\right) \le \phi \left( \chi ,T\right) + 1. \end{aligned}$$

### Proof

Consider a branch *b* in *T*, and suppose it defines a bipartition having respectively $B_l(b), W_l(b), B_h(b), W_h(b)$ black and white tips on its light and heavy sides. We know from the definition that

2 $$\begin{aligned} \delta \left( \chi ,b\right) = \min \{W_l + B_h, W_h + B_l\}. \end{aligned}$$

Suppose, without loss of generality, that the minimum is $W_l+B_h$. We will now look at the parsimony score for this bicoloration. We put a character change at each of the $W_l$ white leaves on the light side of *b* and at each of the $B_h$ black leaves on its heavy side (*parents* take the opposite color). Then the internal nodes on the light side are colored in black and those on the heavy side in white. The number of color changes of this extension is $W_l+B_h+1$ because we have to add an extra change at branch *b*. Since the parsimony score of the bicoloration is the minimum taken over all the possible extensions, we have that

$$\begin{aligned} \text {ps}\left( \chi ,T\right) \le W_l+B_h+1 = \delta \left( \chi ,b\right) +1. \end{aligned}$$

Since this is true for any branch $b\in T$, we obtain the announced result. $\square$

### Asymptotic results for fixed *p*

In this subsection, we use inequality (1) between the parsimony score and the transfer index to establish that the transfer index converges to $p-1$ when *p* is fixed and *n* grows to infinity. Let us consider a random bicoloration $\chi _p$ from ${\mathcal {X}}_p$. Let *T* be a given binary phylogenetic tree with *n* tips colored by $\chi _p$. The larger *n*, the more dispersed the black tips in *T*, and the higher the probability that the parsimony score is equal to *p* and the transfer index to $p-1$. This is formalized as follows.

#### Proposition 1

Let *T* be a given binary phylogenetic tree with *n* tips, and $\chi _p$ be a random bicoloration of the tips of *T*, uniformly chosen from ${\mathcal {X}}_p$. We have that

$$\begin{aligned} {\mathbb {P}}\left( \phi \left( \chi _p,T\right) =p-1\right) \ge {\mathbb {P}}\left( \text {ps}\left( \chi _p,T\right) =p\right) \ge 1 - 4n \times \dfrac{p(p-1)}{n(n-1)}. \end{aligned}$$

#### Proof

The first inequality is an obvious consequence of inequality (1) and upper bound $p-1$ on the transfer index. To demonstrate the second inequality, we use a result by (Steel and Penny 2005, Proposition 9.4.1), establishing that if the color (state) changes in a tree are rare enough that any two edges with changes are separated by at least three edges with no changes, then the parsimony score is guaranteed to coincide exactly with the number of changes within the tree. Since $\chi _p$ contains *p* black tips, if we color all internal nodes white, we will have *p* changes on the external edges leading to the black tips. If the number of edges separating any pair of two black tips is at least 5, then the parsimony score $\text {ps}\left( \chi _p,T\right)$ is equal to *p*, as all changes are separated by at least 3 internal edges with white vertices at both ends. Now, recall that the tree *T* is fixed, but the bicoloration $\chi _p$ is uniformly chosen from ${\mathcal {X}}_p$. Thus, we have:

$$\begin{aligned}&{\mathbb {P}}\left( \text {ps}\left( \chi _p,T\right) = p \right) \ge {\mathbb {P}}\left( \text {any pair of black tips from } \chi _p \text { is at distance} \ge 5 \text { in } T\right) \\&\quad \ge 1- {\mathbb {P}}\left( \text {at least one pair of black tips from } \chi _p \text { is at distance} \le 4 \text { in } T \right) . \end{aligned}$$

For any given tip *l* in the (binary) tree *T*, the number of tips that are separated from *l* by 4 edges or less is at most 8 ($2^{k-1}$ where *k* is the edge distance, corresponding to a fully balanced binary tree of depth *k* with its root connected to *l*), and thus the number of pairs of tips at a distance of 4 edges or less is at most 4*n*. Moreover, as we have $p(p-1)/2$ pairs of black tips among $n(n-1)/2$ pairs of tips in total, the probability for a given pair to contain two black tips is equal to $p(p-1)/n(n-1)$. We obtain the desired inequality by combining these two statements using the union bound. $\square$

This result is valid for any given binary phylogenetic tree *T* as long as the bicoloration $\chi _p$ is uniformly distributed in the set ${\mathcal {X}}_p$. It has the following immediate consequences.

#### Corollary 1

For any given tree *T*, and any $\chi _p$ uniformly distributed in the set ${\mathcal {X}}_p$, we have that,

- when *p* is fixed, the transfer index $\phi (\chi _p,T)$ converges in probability to $p-1$ when $n\rightarrow \infty$;
- when $p=o(\sqrt{n})$, we have that $\phi (\chi _p,T) - (p-1)$ converges in probability to 0 when $n\rightarrow \infty$.

## Behavior of the transfer distance when *p* grows with *n*

In the previous section, we showed that, when *n* tends to infinity, the transfer index converges in probability to $p-1$ for fixed *p*, and TS converges to 0 when *p* grows slowly as $o(\sqrt{n})$. However, simulations in Fig. 1 suggest that the transfer index also behaves in a similar manner for larger values of *p* relative to *n*. For example, for all null models when $p = {\lfloor }{n/2}{\rfloor }$, the expected value of TS is larger than 0.1 with $n = 128$, but lower than 0.05 with $n = 1024$. In this section, we will show that for “all values of *p*”, the distribution of the transfer index is *concentrated* around $p-1$, meaning that the probability of the transfer index being “far away” from $p-1$ vanishes as *n* grows. This explains what we observe in our simulations and motivates the use of $p-1$ as the normalization term in the definition of TS.

The results we obtain in this section are based on concentration inequalities. More precisely, we make use of the Chernoff-Hoeffding bounds for sums of independent random variables, as stated by Dubhashi and Panconesi (2009). In his original paper, Hoeffding (1963) proved that these inequalities also hold for sums of variables obtained by sampling without replacement, which is the case of interest here. The following lemma is a direct consequence of the results in Hoeffding (1963) and Dubhashi and Panconesi (2009).

### Lemma 2

(Chernoff-Hoeffding bound) Let $X=\sum _{i=1}^m X_i$ where $(X_i)_{1\le i\le m}$ denotes a random sample drawn without replacement from a finite set of values in [0, 1]. Then, for any $r>0$

3 $$\begin{aligned} {\mathbb {P}}(X \ge (1+r){\mathbf {E}}[X]) \le \left( \frac{e^r}{(1+r)^{(1+r)}}\right) ^{{\mathbf {E}}[X]} \end{aligned}$$

and for any $t>0$, we have

4 $$\begin{aligned} {\mathbb {P}}(X \ge {\mathbf {E}}[X]+t) \le e^{\frac{-2t^2}{m}}. \end{aligned}$$

We can now state the main theorem of this section.

### Theorem 1

Let *T* be any given binary phylogenetic tree with *n* tips, and let $\chi _p$ be a bicoloration of the tips in *T* chosen uniformly at random from ${\mathcal {X}}_p$. Then, there exists $N\in {\mathbb {N}}$, s.t. for all $n\ge N$, with probability at least $1-O(\frac{1}{n})$,

1. if $p=O(n^{\alpha })$ for some $0< \alpha <1$, then $\phi (\chi _p,T) \ge p-C$ for some constant *C*;
2. if $p=cn + o(n)$ for some $0<c<1/2$, then $\phi (\chi _p,T) \ge p-C\log n$ for some constant *C*;
3. if $p=\frac{1}{2}n-o(n)$, then $\phi (\chi _p,T) \ge p-C\sqrt{n\log n}$ for some constant *C*.

These three cases correspond to different growth rates of *p*, from the slowest (1) to the fastest (3). Case 1 is already partly covered for ${0<\alpha <1/2}$ by Proposition 1 and Corollary 1, which imply that for these values of *p*,

$$\begin{aligned} {\mathbb {P}}\left( \phi (\chi _p,T) = p -1 \right) \ge 1 - O\left( \frac{1}{n^{1-2\alpha }} \right) . \end{aligned}$$

Case 2 corresponds to what we observe in Fig. 1 [see also Extended Data Fig. 1 in Lemoine et al. (2018)], where for any given ratio *p* / *n* (e.g. $p/n = 1/4$), the expected value of the TS decreases when *n* increases. In Case 3, *p* is as large as possible, and the difference between $\phi$ and $p-1$ is the largest among all three cases, as shown in Fig. 1. Note that the bound in Case 3 also holds when $p = n/2$ when *n* is even and $p=n/2-1$ when *n* is odd.

### Corollary 2

For any given tree *T*, any $\chi _p$ uniformly distributed in the set ${\mathcal {X}}_p$, and any *p* that grows with *n* as in cases 1, 2, and 3, the transfer support $\text {TS}(\chi _p,T)$ converges in probability to 0 when $n\rightarrow \infty$.

### Remark 1

The convergence established by the previous corollary also holds for TBE, which is obtained by averaging TS over *N* “bootstrap trees”, when all those trees follow the same null model.

### Proof of Theorem 1

Consider a given bipartition $b \in T$ and let $s \in \left\{ l,h\right\}$ denote the light/heavy side of *b*. Let $q_{s}(b)$ be the number of taxa on side *s* of *b*, which is deterministic since it depends only on *b*. Let $B_s(b)$ be the number of black taxa on side *s* of *b*. Notice that $B_s(b)$ is a random variable since it depends not only on *b*, but also on the bicoloration $\chi _p$. Then, the transfer distance between *b* and the bicoloration $\chi _p$ can be written as

$$\begin{aligned} \delta (\chi _p,b)=\min \left\{ p+q_{l}(b)-2B_l(b),p+q_{h}(b)-2B_h(b) \right\} . \end{aligned}$$

Consequently, we can write the transfer index as

$$\begin{aligned} \phi (\chi _p,T)=\min _{b \in T,s \in \left\{ l,h\right\} } p+q_{s}(b)-2B_s(b). \end{aligned}$$

For any $1<u<p$, define ${\mathcal {B}}_u=\left\{ b \in T: q_{l}(b) \ge u \right\}$, the (deterministic) set of bipartitions in *T* with at least *u* tips on both sides. We are interested in the set ${\mathcal {B}}_u$ since only the bipartitions in this set can give $\delta (\chi _p,b) \le p-u$. This statement derives from some simple arguments based on the definition of the transfer distance. Consider any $b'\notin {\mathcal {B}}_u$, we necessarily have $q_{l}(b')<u<p<q_{b'h}$, $0\le B_l(b')\le q_{l}(b')$, and $0\le B_h(b')\le p$, which implies that

$$\begin{aligned} p + q_{l}(b') -2B_l(b')&\ge p - q_{l}(b')> p - u, \\ p + q_{h}(b') - 2 B_h(b')&\ge q_{h}(b') - p> p - q_{l}(b') > p - u. \end{aligned}$$

As a consequence, we can bound the tail probability of the transfer index using the union bound over all bipartitions in ${\mathcal {B}}_u$, that is

5 $$\begin{aligned} {\mathbb {P}}\left( \phi (\chi _p,T) \le p-u\right)&\le \sum _{b \in {\mathcal {B}}_u} {\mathbb {P}}\left( \delta (\chi _p,b) \le p-u\right) \nonumber \\&\le \sum _{b \in {\mathcal {B}}_u, s\in \{l,h\}} {\mathbb {P}}\left( B_s(b) \ge (q_{s}(b)+u)/2\right) . \end{aligned}$$

We will now derive a bound on each of the elements of the above sum. First, notice that every $B_s(b)$ can be written as

$$\begin{aligned} B_s(b)=\sum _{i=1}^{q_{s}(b)} X_{bsi}, \end{aligned}$$

where $X_{bsi}$ are Bernoulli random variables being equal to 1 if the *i*-th leaf on side *s* of *b* is colored black and 0 otherwise. Thus, $B_s(b)$ follows a hypergeometric distribution and we have

$$\begin{aligned} {\mathbb {E}}\left[ B_s(b)\right] =\frac{pq_{s}(b)}{n}. \end{aligned}$$

Moreover, Chernoff-Hoeffding inequalities (3) and (4) apply to these hypergeometric variables and enable us to derive the appropriate bounds.

We now must consider three cases depending on the growth rate of *p* with respect to *n*.

**Case 1:**$p=O(n^\alpha )$ for some $\alpha <1$.

Applying the bound in inequality (3) with $r = n\left( q_{s}(b)+u\right) /\left( 2pq_{s}(b)\right) -1$, we get

6 $$\begin{aligned}&{\mathbb {P}}\left( B_s(b) \ge \dfrac{q_{s}(b)+u}{2}\right) \le \left( \frac{e^{\frac{n(q_{s}(b)+u)}{2pq_{s}(b)}-1}}{\left( \frac{n(q_{s}(b)+u)}{2pq_{s}(b)}\right) ^{\frac{n(q_{s}(b)+u)}{2pq_{s}(b)}}}\right) ^{\frac{pq_{s}(b)}{n}} \nonumber \\&\qquad \ \le \exp \left( \frac{pq_{s}(b)}{n} \left( \frac{n(q_{s}(b)+u)}{2pq_{s}(b)}-1-\frac{n(q_{s}(b)+u)}{2pq_{s}(b)}\log \frac{n(q_{s}(b)+u)}{2pq_{s}(b)}\right) \right) \nonumber \\&\qquad \ \le \exp \left( \frac{q_{s}(b)+u}{2}-\frac{pq_{s}(b)}{n}-\frac{q_{s}(b)+u}{2}\log \frac{n(q_{s}(b)+u)}{2pq_{s}(b)}\right) \nonumber \\&\qquad \ \le \exp \left( -\frac{q_{s}(b)+u}{2}\left( \log n - \log p + \log \frac{(q_{s}(b)+u)}{2q_{s}(b)}-1\right) -\frac{pq_{s}(b)}{n}\right) . \end{aligned}$$

Since $p=O(n^\alpha )$ for some $0<\alpha <1$, there exist some $N_1\in {\mathbb {N}}$ and $A>0$ such that $p\le An^{\alpha }, \forall n\ge N_1$. This implies that for $n\ge N_1$, we have $\log n - \log p \ge (1-\alpha )\log n - \log A$, which, used in inequality (6), gives

$$\begin{aligned}&{\mathbb {P}}\left( B_s(b) \ge (q_{s}(b)+u)/2\right) \\&\quad \le \exp \left( -\frac{q_{s}(b)+u}{2} \left( (1-\alpha )\log n + \log \frac{(q_{s}(b)+u)}{2q_{s}(b)A}-1\right) -\frac{pq_{s}(b)}{n}\right) . \end{aligned}$$

Using the fact that $q_{s}(b) \ge u$ for any $b \in {\mathcal {B}}_u$, we obtain

$$\begin{aligned} {\mathbb {P}}\left( B_s(b) \ge (q_{s}(b)+u)/2\right) \le \exp \left( -u \left( (1-\alpha )\log n -\log (2A) -1\right) \right) . \end{aligned}$$

Taking $u = 2/(1-\alpha )$ and using inequality (5), we obtain

$$\begin{aligned} {\mathbb {P}}\left( \phi (\chi _p,T)< p-\frac{2}{1-\alpha } \right)&\le \sum _{b \in {\mathcal {B}}_{2/(1-\alpha )}, s\in \{l,h\}} \exp \left( -2\log n +const\right) \\&< 2n \frac{g}{n^2}=\frac{2g}{n}, \end{aligned}$$

where *g* is a constant and we used the fact that $|{\mathcal {B}}_{u}\times \{l,h\}|<2n$ for any *u*. It follows that ${\mathbb {P}}(\phi (\chi _p,T) \ge p-2/(1-\alpha )) >1-O(\frac{1}{n})$ as required.

**Case 2:**$p=cn+o(n)$ for some $0<c<\frac{1}{2}$.

Applying inequality (4) with $t=(q_{s}(b)+u)/2-pq_{s}(b)/n=q_{s}(b)(\frac{1}{2}-\frac{p}{n})+\frac{u}{2}$, we obtain

7 $$\begin{aligned} {\mathbb {P}}\left( B_s(b) \ge \frac{q_b+u}{2}\right)&\le \exp \left( -2\left[ q_{s}(b) \left( \frac{1}{2}-\frac{p}{n}\right) +\frac{u}{2}\right] ^2/q_{s}(b)\right) \nonumber \\&= \exp \left( -2\frac{q^2_{s}(b)(\frac{1}{2}-\frac{p}{n})^2+2q_{s}(b)(\frac{1}{2}-\frac{p}{n})\frac{u}{2}+\frac{u^2}{4}}{q_{s}(b)}\right) \nonumber \\&= \exp \left( -2q_{s}(b) \left( \frac{1}{2}-\frac{p}{n}\right) ^2-2\left( \frac{1}{2}-\frac{p}{n}\right) u-\frac{u^2}{2q_{s}(b)}\right) . \end{aligned}$$

Dropping the last term in the exponent and again using the fact that $q_{s}(b) \ge u$, we obtain

$$\begin{aligned} {\mathbb {P}}\left( B_s(b) \ge (q_{s}(b)+u)/2\right)&\le \exp \left( -2q_{s}(b) \left( \frac{1}{2}-\frac{p}{n}\right) ^2-2\left( \frac{1}{2}-\frac{p}{n}\right) u\right) \\&\le \exp \left( -2u\left[ \left( \frac{1}{2}-\frac{p}{n}\right) ^2+\left( \frac{1}{2}-\frac{p}{n}\right) \right] \right) . \end{aligned}$$

Recall that $p=cn + o(n)$. Let $c<c+\epsilon <1/2$. For some $N_2\in {\mathbb {N}}$, we will have that $p/n<c+\epsilon$ for all $n\ge N_2$, so we can take $u=C \log n$, with $C=\left[ (\frac{1}{2}-(c+\epsilon ))^2+(\frac{1}{2}-(c+\epsilon ))\right] ^{-1}$ and use Eq. (5) to obtain that

$$\begin{aligned} {\mathbb {P}}\left( \phi (\chi _p,T)< p - C \log n\right)< 2n\exp (-2\log n) < \frac{2}{n}, \end{aligned}$$

which gives ${\mathbb {P}}(\phi (\chi _p,T) \ge p - C\log n) > 1-O(\frac{1}{n})$ as required.

**Case 3:** $$p=\frac{1}{2}n-o(n)$$

Since $\frac{p}{n} \rightarrow \frac{1}{2}$ as $n \rightarrow \infty$, the first two terms in the exponent on the right-hand side of inequality (7) tend to 0, so the bound from the previous case is no longer useful. Based on inequality (7), we can write

$$\begin{aligned} {\mathbb {P}}(B_s(b) \ge (q_{s}(b)+u)/2) \le \exp \left( -\frac{u^2}{2q_{s}(b)}\right) . \end{aligned}$$

Knowing that $q_{s}(b) < n$ for any choice of *b*, we can take $u=C\sqrt{n\log n}$, which gives

$$\begin{aligned} {\mathbb {P}}\left( B_s(b) \ge (q_{s}(b)+C\sqrt{n\log n} )/2\right) < \exp \left( -\frac{C^2n\log n}{2n}\right) = \exp \left( -\frac{1}{2}C^2\log n\right) . \end{aligned}$$

Setting $C=2$ and using Eq. (5), we get

$$\begin{aligned} {\mathbb {P}}\left( \phi (\chi _p,T)< p - 2\sqrt{n\log n} \right) < 2n\exp (-2\log n)=\frac{2}{n}, \end{aligned}$$

which gives us the result. $\square$

## Exact distribution of the transfer index on caterpillar trees

In this section, we provide exact formulae for the transfer index distribution on caterpillar trees. We shall see in the discussion section that these formulae can be used to compute p-values for the general case, under suitable assumptions (conjectures). Moreover, the combinatorial techniques used here could potentially help obtain similar results with other kinds of trees (e.g. fully balanced).

**Fig. 3:**
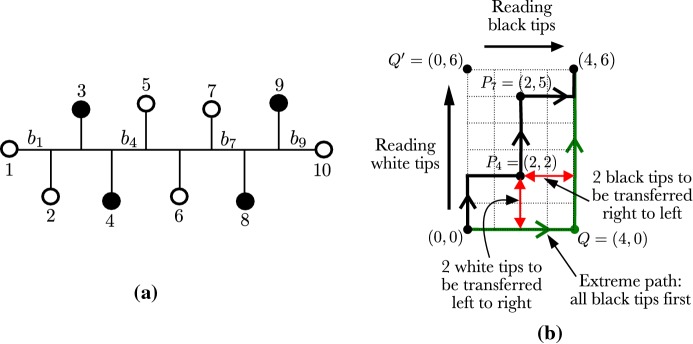
**a** A bicolored oriented caterpillar tree on $$n=10$$ tips with $$p=4$$; tips are numbered from 1 to 10, and branches on the path from tip 1 to tip 10 are denoted $$b_{1}$$ to $$b_{9}$$. **b** The associated (*F* function) NE lattice path from (0, 0) to (4, 6) (bold lines); the points $$P_4=(2,2)$$ and $$P_7=(2,5)$$, on the path, correspond to branches $$b_4$$ and $$b_7$$, on the tree; the green path represents the extreme lattice path corresponding to the bicoloration of black tips first, then white tips; $$Q'$$ corresponds to the opposite: white first, then black. In red the horizontal and vertical distances between $$P_4$$ and *Q* (i.e. the Manhattan distance, $$= 4$$), which corresponds to the number of tips to be transferred from one side of $$b_4$$ to the other (distance between $$P_4$$ and $$Q'$$$$=6$$). The closest point in the path to any of the two corners *Q* and $$Q'$$ is $$P_7$$ (distance to $$Q' = 3$$), which corresponds to the transfer index of the bicoloration in **a** and the move of 2 black and 1 white tips across branch $$b_7$$ (color figure online)

As a reminder, a caterpillar tree is a binary phylogenetic tree for which the induced subtree on the interior vertices forms a path graph (see Fig. 3, left). A cherry is a pair of adjacent tips on a tree. There is a single unlabeled topology for a caterpillar tree with *n* leaves. To identify the leaves conveniently, we label them using the natural ordering induced by the caterpillar tree topology. The tips in the two cherries have labels 1, 2, $n-1$ and *n*, and the other tips are labeled accordingly (Fig. 3, left). In what follows, we use *T* to denote the caterpillar tree labeled in that manner. This labeling/ordering is not unique, but the results are independent of the labeling options for the cherries. Since we study the distribution of the transfer index $\phi (\chi _p,T)$ where $\chi _p$ is uniformly chosen from ${\mathcal {X}}_p$, all bicolorations are equally probable, and our results remain identical with other labeling options. We call the tree *T* endowed with such labeling and coloration a bicolored oriented caterpillar tree.

### Correspondence between bicolored caterpillar trees and NE lattice paths

A *NE lattice path* is a path in ${\mathbb {Z}}^2$ where the only steps allowed are (0, 1) (a step towards the north) and (1, 0) (a step towards the east). From now on, we call them lattice paths for short. Let ${\mathcal {P}}(p,n-p)$ denote the $p \times (n-p)$ NE lattice, that is the set of all lattice paths from the origin (0, 0) to the destination $(p, n-p)$. A path in ${\mathcal {P}}(p,n-p)$ can be encoded in a single vector of length *n* indicating the sequence of steps of the path, which is an element on $\{N,E\}^n$ with a total number of east steps equal to *p* and a total number of north steps equal to $n-p$. On the other hand, the set ${\mathcal {X}}_p$ of bicolorations with *p* black tips is a subset of $\{{\mathbf{B }},{\mathbf{W }}\}^n$. We define the function $F: {\mathcal {X}}_p \rightarrow {\mathcal {P}}(p,n-p)$ that associates a lattice path to a bicolored tree by scanning the tips on the tree from 1 to *n* as follows: whenever we read a white tip, we move towards the north; and whenever we read a black tip, we move towards the east. Consequently, a bicolored oriented caterpillar tree on *n* tips with *p* black tips, corresponds to a unique path in ${\mathcal {P}}(p,n-p)$ and vice versa, as represented in Fig. 3. This result can be summarized as follows.

#### Lemma 3

The function *F* is a bijection from ${\mathcal {X}}_p$ to ${\mathcal {P}} (p,n-p)$.

Let us denote the lower right corner in ${\mathcal {P}} (p,n-p)$ by $Q=(p,0)$ and the upper left corner by $Q'= (0,n-p)$. Observe that the two extreme paths going through *Q* and $Q'$ correspond to the only bicolored oriented caterpillar trees *T* with transfer index $\phi (\chi _p,T) = 0$: all black leaves cluster on one side and all white leaves on the other side (green path in Fig. 3). Moreover, we are able to retrieve the transfer index for any bicolored oriented caterpillar tree from the associated lattice path, as we demonstrate in the following proposition. Use *M*(*A*, *B*) to denote the Manhattan distance between any two lattice points $A,B\in {\mathbb {Z}}^2$, and by $M(\gamma ,B) = \min _{A\in \gamma } M(A,B)$ the Manhattan distance between any lattice path $\gamma$ and a lattice point *B*.

#### Proposition 2

Consider an oriented caterpillar tree *T*, a bicoloration $\chi _p\in {\mathcal {X}}_p$ of its tips, and the corresponding path $\gamma \in {\mathcal {P}} (p,n-p)$. We have that

$$\begin{aligned} \phi (\chi _p,T) = \min \left( M\left( \gamma ,Q\right) ,M\left( \gamma ,Q'\right) ,p-1\right) . \end{aligned}$$

#### Proof

Consider an oriented caterpillar tree *T*, a bicoloration $\chi _p$, and the corresponding lattice path $\gamma$ from (0, 0) to $(p,n-p)$. Let us denote the $n-1$ consecutive internal lattice points in $\gamma$ by $P_1 = (x_1,y_1),\ldots , P_{n-1}=(x_{n-1},y_{n-1})$. Also, use $b_{i}$ to denote the internal branch in *T* between tips *i* and $i+1$, for $2\le i\le n-2$. Lastly, let $b_{1}$ and $b_{n-1}$ be the pendant branches of tips 1 and *n* respectively (Fig. 3, left).

In the same manner that *F* associates tips in the bicolored tree with steps in $\gamma$, this function can be extended naturally so it establishes a one-to-one mapping from a set of branches in *T* to the interior lattice points in $\gamma$. More precisely, if we extend *F* to the set of branches in *T*, it holds that $F(b_i)=P_i$, for all $1\le i\le n-1$. The transfer distance between $\chi _p$ and an internal branch $b_{i}$ is the number of tips to be transferred from one side of $b_{i}$ to the other side that results in the bipartition $\chi _p$. Fix $1\le i\le n-1$ and consider the left side of $b_i$. Use $W(b_i)$ and $B(b_i)$ to denote respectively the number of black and white tips in this left side. By construction, we have that $W(b_i)= y_i$ and $B(b_i) = x_i$, which, together with (2), leads to the following identity

$$\begin{aligned} \delta \left( \chi _p, b_i \right)&= \min \left( x_i + n - p - y_i, p - x_i + y_i \right) . \end{aligned}$$

On the other hand, if we look at the Manhattan distance between the corresponding lattice point $P_i$ and the corners *Q* and $Q'$, we have that $M(P_i,Q) = p - x_i + y_i$ and $M(P_i,Q') = x_i + n - p - y_i$ (see Fig. 3). The same argument applies to any branch $b_i$ for $1\le i\le n-1$. Hence, the minimum of these distances, taken over all lattice points $P_i$ in the path $\gamma$, corresponds to the minimum of the transfer distance obtained by any internal branch in *T*, or by the leaves 1 and *n*.

Finally, all branches on the caterpillar tree that are not on the path from leaf 1 to leaf *n*, are pendant branches. The minimum over all the pendant branches is equal to $p-1$, obtained on any black leaf, so the transfer index is at most $p-1$, as stated in the proposition. Also notice that, in the case of a bicolored cherry, the choice of the labels (1 versus 2 and $n-1$ versus *n*) has no influence on the result since the distance from any of these pendant branches to the reference bipartition is at least $p-1$. Since we have covered the distance obtained on any branch of the tree, we achieve the desired result. $\square$

### Counting bicolorations through lattice paths: the transfer index distribution

Lattice paths under certain restrictions appear in various problems in probability and statistics, such as the classical ballot problem (for instance, see Feller (1968)), which leads to counting lattice paths that do not touch the diagonal $y=x$. Here, we are interested in a slightly different problem, in the sense that we count NE lattice paths that are not allowed to touch certain boundaries.

More precisely, for fixed *n* and *p*, consider $2\le l\le p+1$ and let ${\mathscr {L}}(n,p,l)$ denote the subset of paths in ${\mathcal {P}} (p,n-p)$ that do not touch $y = x - l$ or $y = x + (n-2p+l)$. Set $L(n,p,l):=|{\mathscr {L}}(n,p,l)|$. The following result is from Mohanty (1979). Here, we will give a sketch of the proof that is slightly different from the one given by Mohanty (1979), since it will be useful for understanding the upcoming results. We use ${\lfloor }{\cdot }{\rfloor }$ and ${\lceil }{\cdot }{\rceil }$ to denote respectively floor and ceiling functions, and $\mathbb {1}(\cdot )$ for the indicator function.

#### Lemma 4

(Mohanty, 1979) Let $c = n - 2p + 2l$, then

$$\begin{aligned} L(n,p,l) = \sum \limits _{k = {\lfloor }{\frac{p-l-n}{c}}{\rfloor }}^{{\lceil }{\frac{p}{c}}{\rceil }} \left[ \left( {\begin{array}{c}n\\ p-kc\end{array}}\right) - \left( {\begin{array}{c}n\\ p-l-kc\end{array}}\right) \right] . \end{aligned}$$

#### Proof

The proof is based on André’s reflection method (André 1887). First, notice that a path is uniquely defined by the *p* east steps it makes (or equivalently the $n-p$ north steps), which entails that

$$\begin{aligned} |{\mathcal {P}} (p,n-p)| = \left( {\begin{array}{c}n\\ p\end{array}}\right) . \end{aligned}$$

Let us count now the paths in ${\mathcal {P}} (p,n-p)$ that touch the line $y = x - l$. If a path touching the line $y = x - l$ is reflected from the moment it first touches this line, by switching north steps into east steps and vice versa, we obtain a lattice path ending at $(n-p+l,p-l)$. In fact, this reflection yields a bijection between the set of paths in ${\mathcal {P}} (p,n-p)$ touching the line $y = x - l$ and the set ${\mathcal {P}} (n-p+l,p-l)$, both having $$\left( {\begin{array}{c}n\\ p-l\end{array}}\right) $$ elements.

**Fig. 4:**
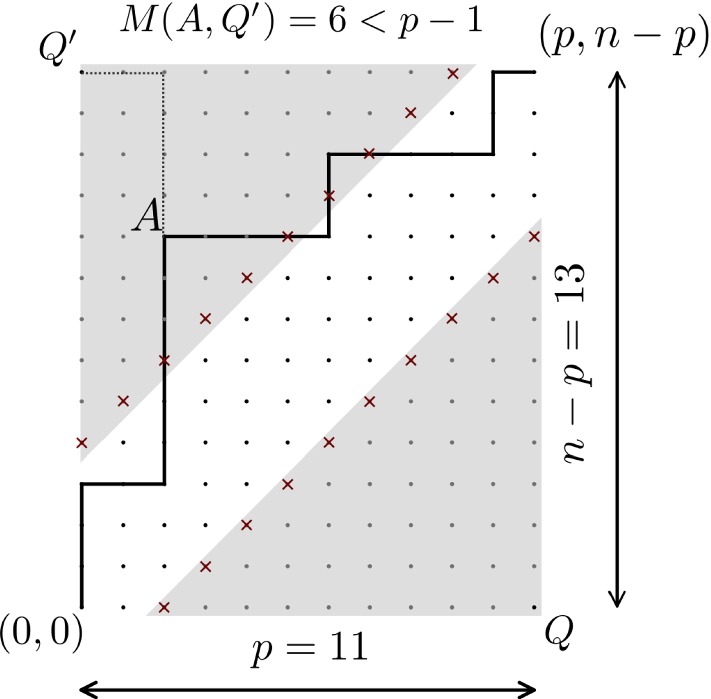
Another example of NE lattice rectangle, with $n=24, \ p = 11$. The lines limiting the shaded areas are $y = x-2$ and $y = x + 4$. Paths avoiding the shaded areas have a Manhattan distance to the corners *Q* and $Q'$ larger than $p-1$. Thus, in the corresponding bicolored caterpillar tree, any internal branch yields a transfer distance larger than $p-1$. However, the transfer index for such a tree is equal to $p-1$, as obtained by any pendant branch leading to a black tip. When a path enters a shaded area, its Manhattan distance to the corners is less than $p-1$, e.g. point *A* is at distance 6 of $Q'$ and thus the corresponding path and bicoloration have a transfer index equal to 6

Likewise, paths in ${\mathcal {P}} (p,n-p)$ that touch the line $y = x + (n-2p+l)$ can be transformed bijectively into paths in the set ${\mathcal {P}} (p-l,n-p+l)$. Then, by applying the *inclusion-exclusion principle* (Fréchet 1940), we have the following identity,

8 $$\begin{aligned} L(n,p,l) = \left( {\begin{array}{c}n\\ p\end{array}}\right) - 2 \left( {\begin{array}{c}n\\ p-l\end{array}}\right) + |{\mathcal {L}}'(n,p,l)|, \end{aligned}$$

where ${\mathcal {L}}'(n,p,l)$ is the set of paths that touch both lines $y = x - l$ and $y = x + (n-2p+l)$. We can then apply the reflection principle repeatedly and the usual inclusion-exclusion principle to account for paths touching both lines multiple times, leading to the above formula after some simplifications. See Mohanty (1979) for further details. $\square$

We can now establish the main theorem in this section.

#### Theorem 2

Let *T* be an oriented caterpillar tree and $\chi _p$ a bicoloration uniformly chosen from the set ${\mathcal {X}}_p$. We have for any $2\le l\le p+1$ that

9 $$\begin{aligned} {\mathbb {P}}\left( \phi \left( \chi _p,T \right) = p-l+1 \right) = \dfrac{L(n,p,l) - L(n,p,l-1) \mathbb {1}(l>2)}{\left( {\begin{array}{c}n\\ p\end{array}}\right) } \end{aligned}$$

and

10 $$\begin{aligned} {\mathbb {E}}\left[ \phi \left( \chi _p,T \right) \right] = \dfrac{1}{\left( {\begin{array}{c}n\\ p\end{array}}\right) }\sum \limits _{l=2}^{p}L(n,p,l). \end{aligned}$$

#### Proof

It is quite straightforward from the definition of ${\mathscr {L}}(n,p,l)$ and Proposition 2 that for any $2\le l\le p+1$, the paths avoiding lines $y = x - l$ and $y = x + (n-2p+l)$ are exactly those that remain at a distance from the corners *Q* and $Q'$ greater or equal to $p-l+1$, as it is shown in Fig. 4, for $l=2$. On the other hand, the paths that do touch these lines give a distance strictly smaller than $p-l+1$. Hence, for $2\le l\le p+1$, a path in the set ${\mathscr {L}}(n,p,l)\setminus {\mathscr {L}}(n,p,l-1)$ (with the convention ${\mathscr {L}}(n,p,1)=\emptyset$) gives a distance exactly equal to $p-l+1$. Since the total number of paths in ${\mathcal {P}} (p,n-p)$ is $$\left( {\begin{array}{c}n\\ p\end{array}}\right) $$, the identity (9) holds. Then, the expectation of the transfer index can be expressed as follows

$$\begin{aligned} E\left[ \phi \left( \chi _p,T \right) \right] = \dfrac{1}{\left( {\begin{array}{c}n\\ p\end{array}}\right) }\sum \limits _{l=2}^{p+1}\left[ L(n,p,l) - L(n,p,l-1) \mathbb {1}(l>2) \right] (p-l+1), \end{aligned}$$

which can easily be simplified to obtain Eq. (10). $\square$

## Discussion

The results we obtained in Sects. 3 and 4 allow us to characterize the asymptotic behavior of the transfer support when *n* tends towards $\infty$, for various growth rates of *p*, up to $p = {\lfloor }{n/2}{\rfloor }$, and for any given binary phylogenetic tree. These results demonstrate that the normalisation by $p-1$ proposed by Lemoine et al. (2018) is fully justified for large *n* and irrespective of the shape of the inferred tree: in the absence of phylogenetic signal, TBE (i.e. the average of TS over all bootstrap trees) is close to 0. The same holds for the standard Felsenstein’s phylogenetic boostrap, as with “random bootstrap trees” the chance of exactly recovering the reference branch is close to zero, even for small values of *p*. On the other hand, Lemoine et al. (2018) used real and simulated data to show that, in the presence of strong phylogenetic signal, Felsenstein’s supports can be close to zero, whereas TBE reveals the signal and provides high support to branches that are (nearly) correct. Moreover, the TBE values can be interpreted in terms of the proportion of stable versus unstable taxa, and the unstable taxa (e.g. recombinant sequences with virus data) can be identified in further analysis, and studied to explain their phylogenetic instability.

However, the bounds we obtained in Sect. 4 are not sufficiently tight to justify what we observe in simulations in Fig. 1. If we think about the applications, these bounds might not be sufficient to give good estimates for the p-values of the TS and TBE distributions in the absence of phylogenetic signal. We propose two conjectures that would allow us to use the exact results obtained for the caterpillar tree as a proxy for the statistical significance of TS and TBE.

The first conjecture concerns the extreme case $p={\lfloor }{n/2}{\rfloor }$. Based on simulation results (Fig. 1 and Lemoine et al. (2018)) and the proofs in Sect. 4, we believe that for any given phylogeny, the expected value of TS attains its maximum over *p* at ${\lfloor }{n/2}{\rfloor }$. The second conjecture (based on Fig. 1 and other experiments, not shown) is that with $p={\lfloor }{n/2}{\rfloor }$, the expected value of TS is highest for caterpillar trees, among all possible tree topologies.

These two conjectures, combined with the fact that TBE is obtained by averaging TS, and thus has necessarily a small variance compared to its mean (see also Fig. 1), forms the basis of a simple test to reject that a branch support could be obtained by chance.

For instance, consider a tree of 128 tips, a bootstrap study with 100 replicates, and a reference branch with $p = n/2 = 64$. Using the exact results of Sect. 5, we can compute the mean and variance of TS distribution in the caterpillar null model, and apply the Central Limit Theorem to approximate the distribution of TBE; the results show that when TBE is larger than 0.147, the p-value of the null hypothesis/model is less than $10^{-3}$. With 256, 512, and 1024 tips, the corresponding $0.1\%$ confidence levels (for $p=n/2$) are equal to 0.108, 0.078, and 0.057, respectively (see Appendix B for more details). When the number of replicates is large (e.g. 1000), the standard deviation of TBE is nearly null and the $0.1\%$ confidence level is roughly equal to the expected value of TS, as shown in Table 1 in Appendix B. Any branch with TBE support less than or equal to these bounds could have been obtained by chance and might not reflect any phylogenetic signal. On the opposite, standard levels of branch support using TBE (say $>70\%$, following Hillis and Bull (1993)) cannot be observed by chance, and reveal a strong phylogenetic signal in the data, even with small trees. Such high support does not tell us that the branch is “true”, but estimates the number of stable versus unstable taxa, given the reconstruction method used to infer the original and bootstrap trees. As explained by Felsenstein (1985), an inconsistent method (e.g. subject to long branch attraction) can produce erroneous trees with high branch supports (TBE as well as Felsenstein’s bootstrap).

For trees that are not caterpillars, deriving the distribution of the transfer index under random bicolorations appears to be challenging. It would be relevant for both theoretical and applicative reasons to characterize this distribution for a random model such as Yule or PDA, which are the most commonly used in phylogenetics.

## Appendix A: Asymptotics of the transfer index on the caterpillar tree

We obtained an additional result describing the asymptotic behavior of the transfer index when *n* gets large, in the case of caterpillar trees. Similarly to our results in Sects. 3 and 4, the following proposition implies that TS tends to 0 when $n\rightarrow \infty$ for random bicolorations. However, the speed of convergence implied by the proposition below improves the one obtained in Sect. 4. In particular, for $p = {\lfloor }{n/2}{\rfloor }$, Theorem 1 implies that the expectation of TS grows at most as $\sqrt{\log n}/\sqrt{n}$, whereas for the caterpillar tree it is $1/\sqrt{n}$ as we demonstrate below.

### Proposition 3

Let *T* be an oriented caterpillar tree and $\chi _p$ a bicoloration uniformly chosen from the set ${\mathcal {X}}_p$, for $2\le p\le {\lfloor }{n/2}{\rfloor }$. The expected transfer support goes to 0 uniformly on *p*, moreover

11 $$\begin{aligned} \max \limits _{p\le n/2} \left( 1 - \dfrac{{\mathbb {E}}\left[ \phi \left( \chi _p,T \right) \right] }{p-1} \right) = O\left( \dfrac{1}{\sqrt{n}}\right) , \ \text {when} \ n\rightarrow \infty . \end{aligned}$$

### Proof

For any *n*, *p*, *l* with $p\le n/2$ and $2\le l\le p+1$ we have from Eq. (8) that

$$\begin{aligned} L(n,p,l) \ge \left( {\begin{array}{c}n\\ p\end{array}}\right) - 2 \left( {\begin{array}{c}n\\ p-l\end{array}}\right) . \end{aligned}$$

This inequality, together with Eq. (10), leads to

$$\begin{aligned} {\mathbb {E}}\left[ \phi \left( \chi _p,T \right) \right] \ge p-1 - \dfrac{2}{\left( {\begin{array}{c}n\\ p\end{array}}\right) }\sum \limits _{l=2}^{p}\left( {\begin{array}{c}n\\ p-l\end{array}}\right) , \end{aligned}$$

or equivalently,

$$\begin{aligned} {\mathbb {E}}\left[ \text {TS}\left( \chi _p,T\right) \right] = 1 - \dfrac{{\mathbb {E}}\left[ \phi \left( \chi _p,T \right) \right] }{p-1} \le \dfrac{2}{(p-1)\left( {\begin{array}{c}n\\ p\end{array}}\right) }\sum \limits _{l=2}^{p}\left( {\begin{array}{c}n\\ p-l\end{array}}\right) =:G(p). \end{aligned}$$

Let us now prove that *G*(*p*) is an increasing function for $2\le p \le {\lfloor }{n/2}{\rfloor }$,

$$\begin{aligned} G(p+1) - G(p)&= \dfrac{2}{(p)\left( {\begin{array}{c}n\\ p+1\end{array}}\right) }\sum \limits _{k=0}^{p-1}\left( {\begin{array}{c}n\\ k\end{array}}\right) - \dfrac{2}{(p-1)\left( {\begin{array}{c}n\\ p\end{array}}\right) }\sum \limits _{k=0}^{p-2}\left( {\begin{array}{c}n\\ k\end{array}}\right) \\&= 2 \sum \limits _{k=1}^{p-1} \left[ \dfrac{\left( {\begin{array}{c}n\\ k\end{array}}\right) }{p\left( {\begin{array}{c}n\\ p+1\end{array}}\right) } - \dfrac{\left( {\begin{array}{c}n\\ k-1\end{array}}\right) }{(p-1)\left( {\begin{array}{c}n\\ p\end{array}}\right) }\right] + \dfrac{2}{p\left( {\begin{array}{c}n\\ p+1\end{array}}\right) } \\&= 2 \sum \limits _{k=1}^{p-1} \dfrac{(p+1)!(n-p-1)!}{(k-1)!(n-k+1)!} \left[ \dfrac{ n-k+1}{pk}- \dfrac{n-p}{p^2-1} \right] \\&\quad + \dfrac{2}{p\left( {\begin{array}{c}n\\ p+1\end{array}}\right) }, \end{aligned}$$

where the terms between brackets in the sum can be expanded as follows

$$\begin{aligned} \dfrac{ n-k+1}{pk}- \dfrac{n-p}{p^2-1} = \dfrac{n(p^2-kp-1)+p^2+k-1}{pk(p^2-1)}. \end{aligned}$$

It is not hard to see that $p^2-kp-1\ge 0$ for all values of *k* and *p* such that $1\le k\le p-1$, which implies that the previous fraction is always positive. We can then conclude that $G(p+1)-G(p)\ge 0$, so the maximum value of *G* is obtained when $p={\lfloor }{n/2}{\rfloor }$.

For the sake of simplicity, we suppose from now that *n* is even, but an equivalent result is obtained for odd *n* without difficulty. For $p= n/2$, we can use the identity $$\sum _{k=0}^n\left( {\begin{array}{c}n\\ k\end{array}}\right) =2^n$$ and the symmetry of the binomial coefficients $$\left( {\begin{array}{c}n\\ k\end{array}}\right) =\left( {\begin{array}{c}n\\ n-k\end{array}}\right) $$ for all $0\le k\le n$, to simplify the sum in the function *G* as follows,

$$\begin{aligned} \sum \limits _{l=2}^{n/2}\left( {\begin{array}{c}n\\ n/2-l\end{array}}\right) = \sum \limits _{k=0}^{n/2-2}\left( {\begin{array}{c}n\\ k\end{array}}\right) = \dfrac{2^n - 2\left( {\begin{array}{c}n\\ n/2-1\end{array}}\right) - \left( {\begin{array}{c}n\\ {\lfloor }{n/2}{\rfloor }\end{array}}\right) }{2}. \end{aligned}$$

So, for all $2\le p \le n/2$, we have that

$$\begin{aligned} 0\le {\mathbb {E}}\left[ \text {TS}\left( \chi _p,T\right) \right] \le G\left( n/2 \right) = \dfrac{2^n - 2\left( {\begin{array}{c}n\\ n/2-1\end{array}}\right) - \left( {\begin{array}{c}n\\ n/2\end{array}}\right) }{\left( n/2-1\right) \left( {\begin{array}{c}n\\ n/2\end{array}}\right) }. \end{aligned}$$

Let us now look at the asymptotic behavior of this upper bound. The well-known Stirling formula yields the following approximation for central binomial coefficients $$\left( {\begin{array}{c}n\\ n/2\end{array}}\right) \sim \frac{2^{n+\frac{1}{2}}}{\sqrt{\pi n}}$$. Hence

$$\begin{aligned} \dfrac{2^n - 2\left( {\begin{array}{c}n\\ n/2-1\end{array}}\right) - \left( {\begin{array}{c}n\\ n/2\end{array}}\right) }{\left( n/2-1\right) \left( {\begin{array}{c}n\\ n/2\end{array}}\right) } \sim \dfrac{\sqrt{2\pi n}}{n-2} - \dfrac{4n}{(n-2)(n+2)} - \dfrac{2}{n-2} \sim \dfrac{\sqrt{2\pi }}{\sqrt{n}}, \end{aligned}$$

which tends towards 0 when $n\rightarrow \infty$, allowing conclusion of the proof. $\square$

## Appendix B: Central limit theorem to approximate TBE distribution on caterpillar trees

Consider $N\in {\mathbb {N}}$. Let *T* be an oriented caterpillar tree with *n* tips, and let $(\chi ^{(i)}_p)_{i=1,\ldots , N}$ be a set of independent bicolorations distributed as $\chi _p$ (uniformly chosen from ${\mathcal {X}}_p$, as in Theorem 2). Let $\mu = {\mathbb {E}}[\phi (\chi _p,T)]$ and $\sigma = \sqrt{\text {Var} [\phi (\chi _p,T)]}$. Recall the definition of TBE given in the introduction, which is the average of TS over all “bootstrap trees”. In the caterpillar null model, this corresponds to

$$\begin{aligned} \text {TBE} (N) = \dfrac{1}{N}\sum _{i=1}^N \text {TS}(\chi ^{(i)}_p,T) ) = \dfrac{1}{N} \sum _{i=1}^N \left( 1 - \dfrac{\phi (\chi ^{(i)}_p,T) )}{p-1}\right) . \end{aligned}$$

Simple properties of the expectation and variance lead to

$$\begin{aligned} \mu _{\text {TS}} :={\mathbb {E}}[\text {TS} (\chi _p,T)] = 1 - \dfrac{\mu }{p-1}, \ \text {and} \ \sigma _{\text {TS}} :=\sqrt{\text {Var} [\text {TS} (\chi _p,T)]} = \dfrac{\sigma }{p-1}. \end{aligned}$$

We are interested in finding the smallest value $\alpha (N)\in [0,1]$, such that

$$\begin{aligned} {\mathbb {P}}\left( \text {TBE}(N) > \alpha (N) \right) < 10^{-3}. \end{aligned}$$

We apply the Central Limit Theorem (CLT) to approximate the distribution of TBE for sufficiently large *N* (e.g. $N= 100$), leading to the classical equation

$$\begin{aligned} \alpha (N) = \mu _{\text {TS}} + z_{0.999}\times \sigma _{\text {TS}}/\sqrt{N}, \end{aligned}$$

where $$z_{0.999} = 3.09$$ is the quantile of order 0.999 for the standard normal distribution. Using this calculation we computed the values of $$\alpha $$ for different values of *n* (with $$p=n/2)$$) and *N*. The results are displayed in the following Table 1.

**Table 1:** Values of the expectation, standard deviation of TS, and 0.1% confidence levels for TBE (using a CLT approximation), for different values of *n* (with $$p=n/2)$$ and *N*

*n*	$\mu$	$\sigma$	$\alpha (N = 100)$	$\alpha (N = 1000)$
16	0.290	0.147	0.336	0.305
32	0.230	0.098	0.260	0.240
64	0.177	0.067	0.197	0.183
128	0.133	0.047	0.147	0.137
256	0.098	0.033	0.108	0.100
512	0.071	0.023	0.078	0.073
1024	0.051	0.016	0.057	0.053
2048	0.037	0.012	0.041	0.038
4096	0.026	0.008	0.029	0.027
